# Posterolateral Premature Ventricular Complex Manifest as Posteroseptal Premature Ventricular Complex: A Case Report

**DOI:** 10.7759/cureus.48405

**Published:** 2023-11-06

**Authors:** Mohd Khairi Othman, Saravanan Krishinan, Zurkurnai Yusof, W Yus Haniff W Isa

**Affiliations:** 1 Department of Internal Medicine, School of Medical Sciences, Universiti Sains Malaysia, Kubang Kerian, MYS; 2 Cardiology Department, Hospital Sultanah Bahiyah, Alor Setar, MYS; 3 Cardiolgy Unit, Hospital Universiti Sains Malaysia, Kota Bharu, MYS

**Keywords:** radiofrequency ablation, catheter ablation, 3d electrophysiology mapping, high pvc burden, posterolateral pvc

## Abstract

Premature ventricular complex (PVC) is one of the most common arrhythmias detected in young patients. We report a case of a young patient with symptomatic high-burden PVC suspected to originate from the posterior right ventricular outflow tract (RVOT) who underwent an electrophysiology study (EPS) and was subsequently successfully ablated with markedly reduced PVC burden. The following day, it was noted that there was a change in PVC morphology. A repeat 3D electroanatomical mapping localized the second PVC morphology to posterolateral RVOT and abolished it with radiofrequency ablation (RFA).

## Introduction

Premature ventricular complex (PVC) is one of the most common arrhythmias detected in young patients. We report a case of a young patient with symptomatic high-burden PVC suspected to originate from the posteroseptal right ventricular outflow tract (RVOT), who underwent an electrophysiology study (EPS) and shifted to posterolateral PVC RVOT on the following day. The posterolateral PVC RVOT successfully ablated and returned to normal sinus rhythm with difficulty maneuvering the ablation catheter.

## Case presentation

**Figure 1:**
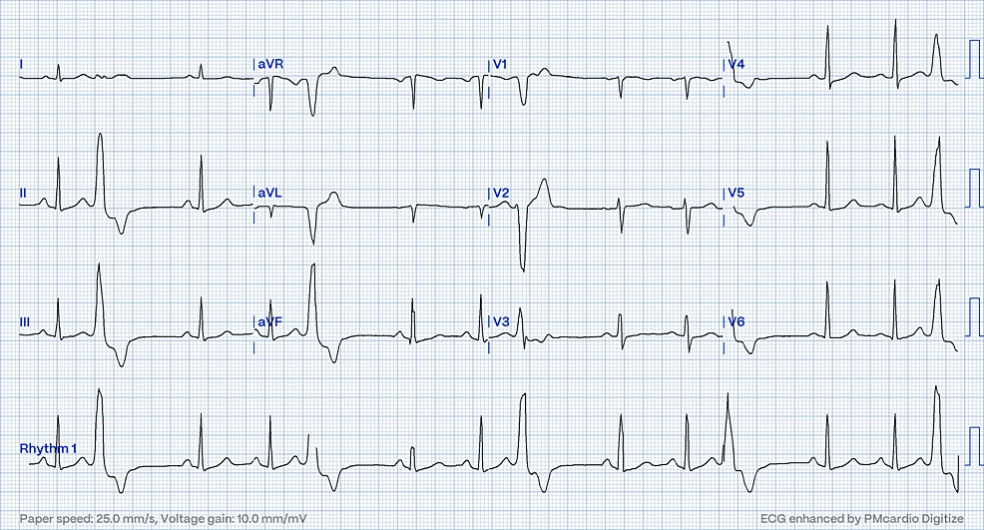
ECG showed PVC with LBBB morphology, the inferior axis with the transition at V3, which suggests the site of the location at posteroseptal RVOT. PVC: premature ventricular complex, LBBB: left bundle branch block, RVOT: right ventricular outflow tract.

A 36-year-old lady with underlying hypertension and dyslipidemia complained of non-progressive intermittent central chest pain radiating to the left upper back for three years. The pain was described as dull, aching, and continuous in nature. She was able to cycle for more than three hours without limitation. She had no symptoms of syncope, acute coronary syndrome, or heart failure in the past. The clinical examination was unremarkable. The ECG is shown in Figure 1.

Twenty-four-hour Holter monitoring showed a unifocal PVC burden of 30%. Echocardiography showed a structurally normal heart. CT coronary angiography showed normal origin and patent coronary arteries. Hence, she was diagnosed with symptomatic unifocal PVC. Based on surface ECG, the PVC had an LBBB morphology with an inferior axis; transitions occurred at V3, rS in lead V1, and an R pattern in V6 suggestive of origin from the posteroseptal RVOT. After the failure of beta-blocker therapy for one year for symptomatic relief, she agreed to undergo EPS and radiofrequency ablation (RFA).

The procedure was done using CARTO 3D mapping with a THERMOCOOL SMARTTOUCH® catheter from Biosense Webster (Irvine, CA). Mapping was done during PVC for the earliest ventricular electrogram (EGM). The earliest activation breakthrough site of PVC was localized to posteriorseptal RVOT and measured 30 ms earlier than the PVC template (Video 1).

**Video 1:** Propagation map showed posteroseptal RVOT origin. RVOT: right ventricular outflow tract.

A pace map was performed, and the PVC match was 93.6%; however, attempted ablation was unsuccessful. RVOT was remapped, and the earlier breakthrough site shifted inferiorly; thus, further lesions were delivered along the same line from the superior to the inferior site within the vicinity of a centimeter. Post-ablation, monitoring the patient for 30 minutes revealed that the PVC frequency was reduced, the procedure was stopped, and the patient was put under observation.

**Figure 2:**
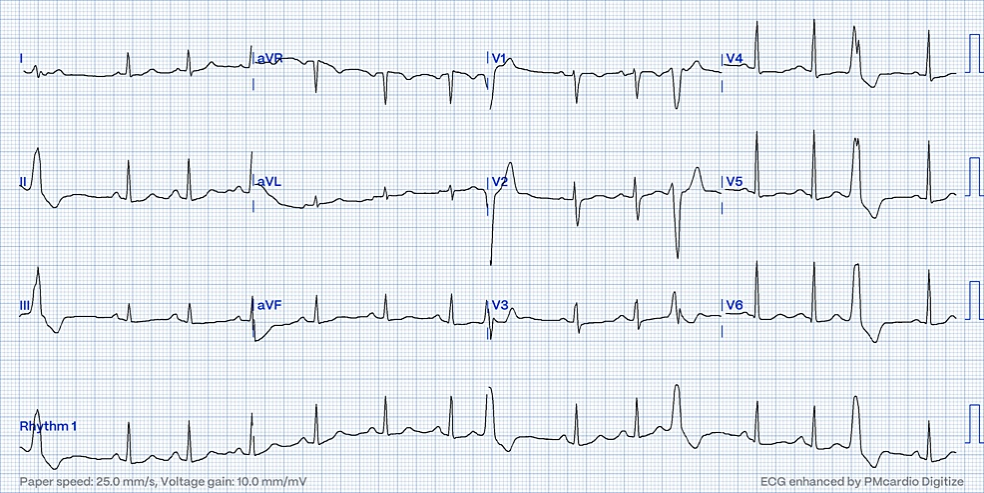
ECG post-first procedure show changed of PVC morphology and transition point.

The next day, a patient had persistent symptoms and documented unifocal PVC of slightly different morphology than the first one, as shown in Figure 2.

The patient consented to repeat the procedure. The coronary cusps were mapped during PVC using the femoral artery approach, but the earliest breakthrough site was 28 ms before surface ECG. The attempted ablation failed to suppress the PVC. Thus, RVOT was remapped. We identified a pre-systolic sharp potential preceding the QRS onset during PVC by 47 ms at the posterolateral aspect of RVOT with 95.9% similarity to the PVC template (Video 2).

**Video 2:** Propagation map showed the PVC site of origin shift to posterolateral RVOT. RVOT: right ventricular outflow tract, PVC: premature ventricular complex.

**Figure 3:**
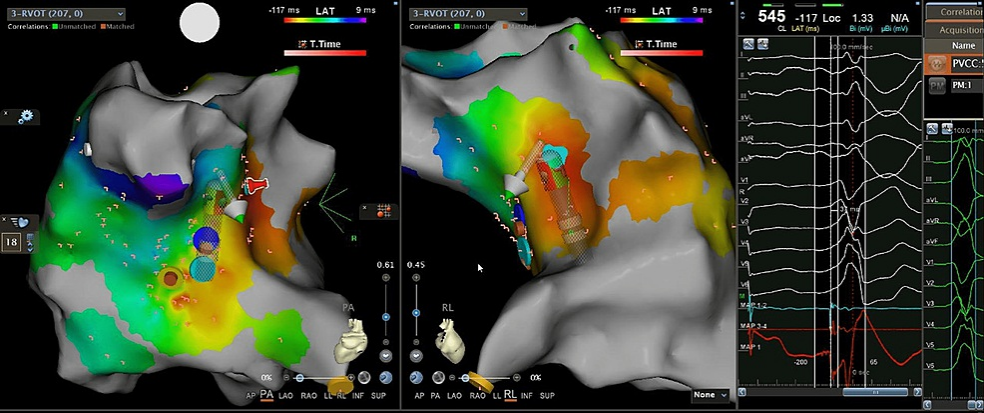
Ablation is done at posterolateral RVOT with 30 W.

This spot is opposite the earliest area identified over the left coronary cusp, as shown in Figure 3.

The PVCs were immediately suppressed with ablation at 30 W in irrigated mode. The lesion was consolidated, and the patient was observed for 30 minutes and showed no recurrence of PVC. There were no complications throughout the procedure. The patient was discharged with a repeated ECG showing sinus rhythm the next day. At the six-month follow-up, the patient was asymptomatic with no documented PVC on the ECG or Holter.

## Discussion

PVC is typically benign in nature; however, in certain cases, such as high PVC burden and tachycardia-induced cardiomyopathy, suppression or termination of PVC is important. Anti-arrhythmia or catheter ablation is the current treatment in patients with high-burden PVC and tachycardia-induced cardiomyopathy. PVC ablation carries low risk and a high success rate, provided the site of origin of the PVC is easily determined pre-procedure and there is only one exit site [1-2]. However, it does not apply to all cases. In our case, we demonstrated the difficulty of ablation of posteroseptal PVC despite baseline ECG suggesting that.

Surface ECG morphology is important to determine the site of origin, as in this patient from RVOT; inferior axis, LBBB with negative QRS complex in V1-V3 and late precordial R/S transition between V3 and V4 [3]. Because of these findings, the initial strategy was to map the RVOT posteroseptal; however, the PVC was unable to suppress. We observed in our patient that the PVC was not suppressed but slightly changed the next day. The final site of origin of this PVC is at the posterolateral RVOT, which is difficult to ablate. The preferential pathway is the most common pathophysiology of change of site of origin in PVC.

Posteroseptal RVOT PVC is more common than posterolateral RVOT PVC; however, it has a higher success rate than posteroseptal origin [4]. A patient with posterolateral PVC may have features of arrhythmogenic right ventricular cardiomyopathy (ARVC), RV cardiomyopathy, and RV non-compaction. Hence, prior to the electrophysiology study in posterolateral PVC, it is important to look for excessive trabeculation in RV, either using echocardiography or cardiac MRI [4]. In our patient, the final site of origin is at the 6-8 o’clock position, as described by Yue-Chun et al., which is the commonest site of posterolateral PVC [5]. Retrospectively, on our patient’s surface, the ECG suggests suggestive depolarization of the myocardium in a direction towards the lead aVL, away from inferior leads, and vertically towards aVR, similar to that described by Das et al. [4].

The main issue in posterolateral PVC ablation is catheter stability during mapping and ablation. It has been reported in the literature that using a long sheath, a superior transjugular approach, or certain maneuvers, such as an anchoring maneuver with flexion of the catheter tip below the ventricular surface of the tricuspid annulus and resting the loop at the junction of the tricuspid annulus and septal leaflet, is recommended to achieve better stability without much manipulation [4]. In our case, we maneuvered the catheter manually based on the activation map and 3D images without using the recommended maneuver but managed to ablate the PVC with a single attempt.

## Conclusions

Identification of the site of origin PVC on surface ECG is important to have a high success rate of ablation. However, the presence of a preferential pathway in our patient contributes to the redo of the procedure in the same hospitalization. In patients who have undergone PVC, extensive planning of a strategy of ablation is necessary to reduce the rate of recurrence.
